# 

**Published:** 1893-05

**Authors:** 


**WHAT IS CHOLERA?**

**By WILLIAM WATSON HALL, M. D.**

10 cts. a copy. MAY, 1893. $1.00 per year.

### HALL'S JOURNAL OF HEALTH

ESTABLISHED 1854

OFFICE, 23 PARK ROW.

Entered as Second-Class Matter at the Post-Office, New York.

